# Impact of sterilization methods on dissolved trace metals concentrations in complex natural samples: Optimization of UV irradiation

**DOI:** 10.1016/j.mex.2019.04.020

**Published:** 2019-04-22

**Authors:** Sandrine Chifflet, Marianne Quéméneur, Aude Barani, Bernard Angeletti, Morgane Didry, Gérald Grégori, Nathalie Pradel

**Affiliations:** a Aix Marseille Univ., Université de Toulon, CNRS, IRD, MIO UM 110, 13288, Marseille, France; b Aix Marseille Univ., CNRS-IRD-Collège de France, CEREGE UM34, 13545, Aix en Provence, France

**Keywords:** UV sterilization of complex natural (lacustrine or coastal marine) samples, UV, Sterilization, Trace metals, Natural ecosystems, Complex matrices

## Abstract

Sterilization is essential for discriminating biotic responses from abiotic reactions in laboratory experiments investigating biogeochemical processes of complex natural samples. However, the conventional methods used to effectively sterilize materials or culture media do not allow sterilizing complex natural samples while maintaining biogeochemical balances. The aim of this study was to develop a low-cost and easy-to-use method to obtain geochemically unmodified and sterilized samples from complex lacustrine or coastal marine ecosystems. In preliminary assays, the impact of several sterilization methods (autoclaving, chemical poisoning, microwave, UV irradiation) on the trace metals balances was studied using borosilicate glass (BG), fluorinated ethylene-propylene (FEP) or polyethylene terephthalate (PET) bottles. Unlike other methods, UV sterilization had minor effects on the distribution of dissolved trace metals. Additional tests using complex lacustrine and coastal marine samples under 10 g/L sediments were performed using a homemade UV sterilization chamber designed to simultaneously irradiate a large number samples. Results showed:

• very reproducible UV tests in BG and FEP bottles
• faster sterilization using FEP bottles than using BG bottles
• low variations of dissolved trace metals concentrations, except for Al, Cu, Fe and Zn

## Graphical abstract

**Figure:**
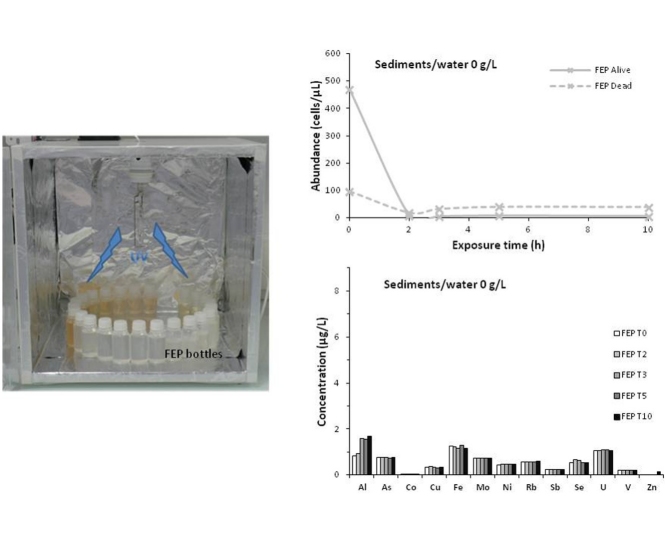

**Specifications Table**

**Table:** 

Subject Area:	Environmental Science
More specific subject area:	Sterilization of natural samples
Method name:	UV sterilization of complex natural (lacustrine or coastal marine) samples
Name and reference of original method:	N/A
Resource availability:	N/A

## Method details

### Background

Trace metals in natural environments are exchanged between different compartments (air, water, sediments) through abiotic and biotic processes [1,2]. In order to quantify and distinguish between the biotic and abiotic reactions involved in biogeochemical cycles, effective abiotic control (i.e. sterilized controls) preventing physico-chemical changes due to biological activities in natural samples are required. Several sterilization techniques have been described in the literature to remove, kill or inactive microorganisms: heat, chemistry, irradiation and filtration [[3], [4], [5]]. All these techniques have advantages and drawbacks (see below as additional background) and need to compromise between the efficiency of the sterilization and the conservation of trace metals distribution, depending on the samples and the scientific objectives. For complex natural samples (such as sediments mixed with water), the major drawback of these sterilization techniques is the possible modification of the sediments structure with a chemical transfer between the solid and liquid phases, modifying the chemical composition of abiotic controls [6,7].

The aim of the present study was to develop a low-cost and easy-to-use method to obtain geochemically unmodified and sterilized controls from complex natural samples (lacustrine or coastal marine ecosystems). In preliminary assays, the impact of the sterilization technique (autoclaving, chemical poisoning, microwave, UV) on the geochemical balances was tested using different moulded bottles made of borosilicate glass (BG), fluorinated ethylene-propylene (FEP) or polyethylene terephthalate (PET). Then, the promising UV sterilization method was further investigated on both lacustrine and marine complex samples (with 10 g/L sediments) by testing the impact of several exposure times (up to 10 h of UV irradiation). Here, we propose a homemade UV sterilization chamber designed to irradiate and obtain simultaneously a large number of abiotic samples. The quantification of viable, damaged or compromised cells within the free-bacterial community was estimated by epifluorescence microscopy observations and monitored by flow cytometry and dissolved trace metals concentrations were measured by ICP-MS.

### Material

**Fig. 1:**
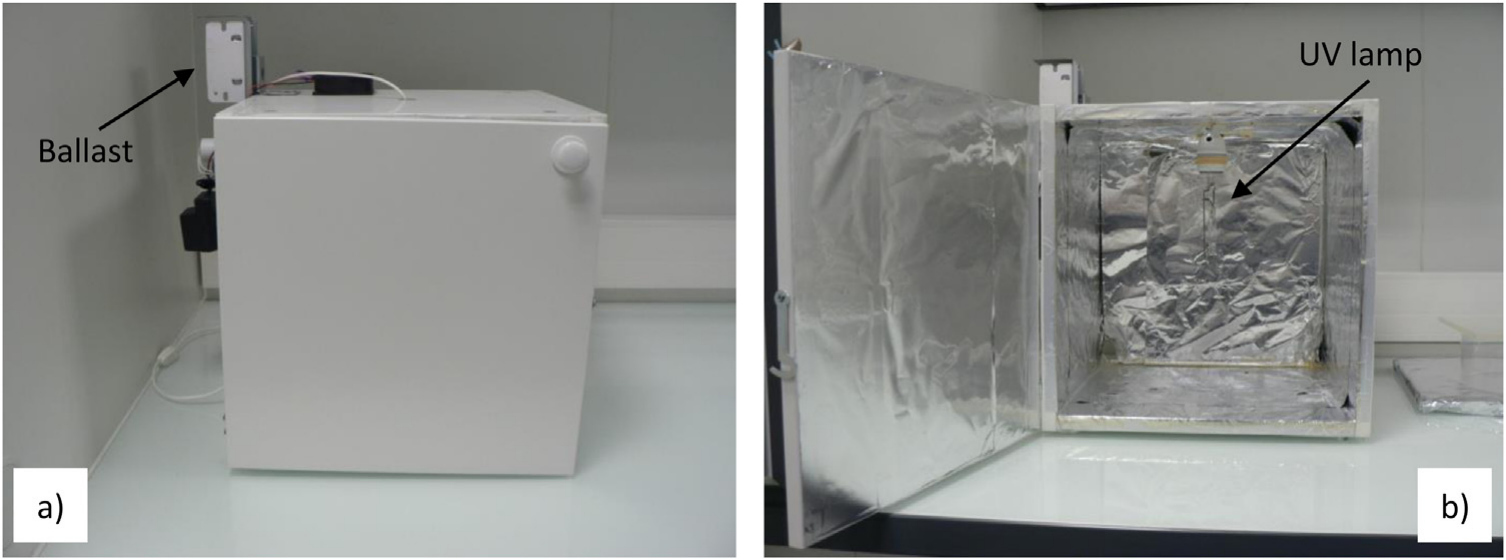
Home-made UV irradiation chamber, lined inside of aluminum foil, equipped with UV lamp (after light bulb was removed) and a ballast for lamp operating. (a) Outside view, (b) Inside view.
• Ultrasonic bath (Branson®, MT 8800-E)
• Chemical reagents
  - NaN3 (Sigma-Aldrich®, ReagentPlus)
  - HCl (VWR®, analytical grade)
  - HCl (Laboratory distillation, ultrapure grade)
  - Decon90 (Decon®, Alkaline detergent)
• Recipients (Fig. 2)
  - For field sampling: 20 L High Density PolyEthylene (Nalgene®, HDPE) jerrycans and 500 mL PolyCarbonate (Nalgene®, PC) bottles
  - For sterilization experiments: 120 mL Borosilicate Glass (Fisherbrand®, BG), PolyEthylene Terephthalate (Thermo Scientific®, PET) or Fluorinated Ethylene-Propylene (Thermo Scientific®, FEP) bottles

• Autoclave (Sanoclav®, 20 L)
• Household microwave oven (LG®, Intellowave)
• Home-made UV irradiation chamber (38 × 38 × 38 cm), lined inside of aluminum foil, equipped with a UV lamp (Philips®, HPL-N 125W) and a ballast (ETL®, VMI 12/23-3) to UV lamp operating (Figs. 1 and S1 as Supplementary information)

**Fig. 2:**
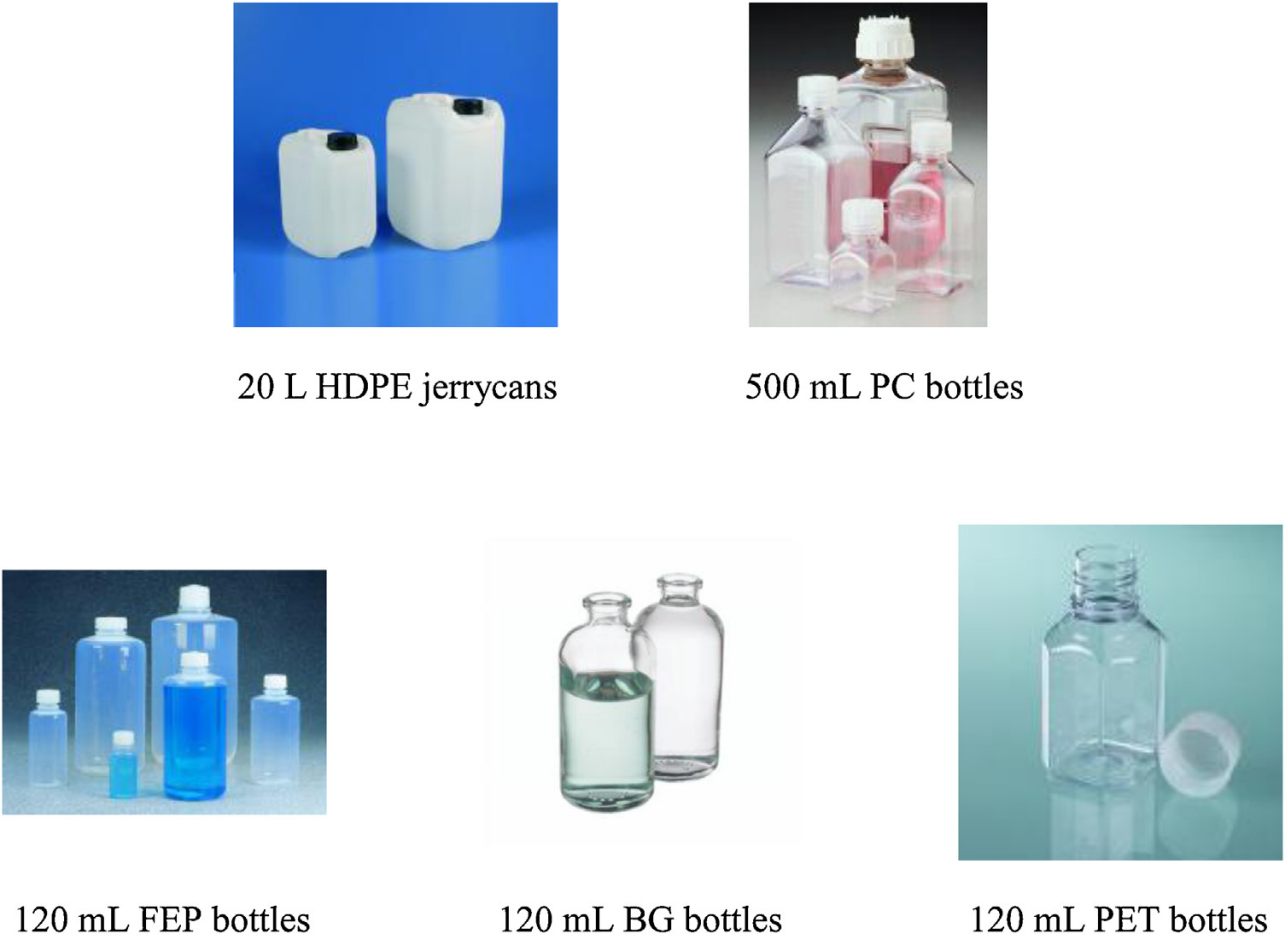
Bottles used for field sample collection and laboratory batch experiments.
• Sterile single-use syringes, luer lock tip (Norm-Ject®, 30 mL)
• Sterile syringe filters (Ministart® High-flow, 0.22 μm)
• Metal free centifuge tubes (VWR®, 15 mL)
• Powder free nitrile gloves (Kimtech®, purple)
• Zip sealed plastic bags for sample storage (Minigrip® bags, various size)
• Pipette (Eppendorf®, 100–1000 μL)
• Pipette tips (Eppendorf®, 1000 μL)

Note: This list does not include analytical laboratory equipment (such as flow cytometer, ICP-MS or microscope) and associated supplies, which are assumed to be available.

### Cleaning procedures

Strict cleaning procedures are essential to avoid contamination by trace metals. The washing lab should be as clean as possible and powder-free nitrile gloves should be worn during all procedures.

1 HDPE jerrycans
  • Fill HDPE jerrycans with Decon for at least 48 h
  • Rinse 5× with deionized water (DIW) or reverse osmosis water (ROW)
  • Fill HDPE jerrycans with HCl (1% v/v, analytical grade) for at least 48 h
  • Rinse 5× with ultra pure water (MilliQ)
  • Store HDPE jerrycans filled with MilliQ until use in a cleaned plastic box or bag
2 BG, PET and FEP bottles
  • Fill and submerse bottles in HCl (10% v/v, analytical grade) bath
  • Sonicate for 2 h or leave in the bath for at least 48 h
  • Rinse 5× with ultra pure water (MilliQ)
  • Store bottles filled with HCl (0.1% v/v, ultrapure grade) until use in zip sealed plastic bags. This last conditioning step must be processed in a trace metal clean-room or under a laminar flow cabinet.

Note: new bottles need to be soaked for at least 48 h in Decon and thoroughly rinsed 7× with DIW or ROW before following the cleaning procedure.

### Field samples collection

Freshwater and associated sediments were manually collected in the Peyrolles lake (France, 44°35’40.253”N - 6°40’10.472”E) from a plastic floating dock. MilliQ in the pre-cleaned HDPE jerrycans was discarded away from site, and then HDPE jerrycans were thoroughly rinsed three times before filling with surface water. Surficial sediments were collected using a Van Veen Grab sampler and transferred into a PC bottle using a plastic spoon. Seawater and coastal sediments were collected in the Bay of Marseille (France, 43°15’23.012”N - 5°22’30.977”E) according to the protocol describe above.

Powder free nitrile gloves were worn during all sampling procedures. Water and sediments samples were kept in the dark at room temperature until use.

### Impact of different sterilization techniques on lacustrine water

The effectiveness of four sterilization techniques (autoclaving, chemical poisoning, microwave, UV) to kill or inhibit the growth of microorganisms while maintaining dissolved trace metals was evaluated on lacustrine water using three types of bottles (BG, FEP, PET) (Table 1). All pre-cleaned bottles were first rinsed three times with an aliquot then filled with 50 mL of the sample. Caps were strongly closed (for most treatment) or loosely closed (for microwave treatment or PET bottles autoclaving) during the sterilization process to avoid any distortion or blasting of the bottles. For each condition (BG, FEP, PET), unsterilized bottles of freshwaters were used as controls to check for the sample evolution (microbial population and the trace metal balances), which can vary according to the container.

**Table 1:** Protocols details used for the sterilization tests.

		Plugging			Protocol
		BG	FEP	PET	
Control		●	●	●	None
Autoclaving		●	●		2 atm, 121 °C, 30 min
Chemical poisoning (NaN_3_)		●	●	●	50 mM
UV		●	●	●	3 h
Microwave	LP			N/A	*160 W, 3 min and dry-ice, 5 min*, repeated 5 times
	MP			N/A	*500 W, 1 min and dry-ice, 5 min*, repeated 5 times

LP: Low Power.

MP: Medium Power.

N/A: Not available.

Strongly closed cap.

● Loosely closed cap.

Autoclaving (heat under stream pressure, 121 °C at a pressure of 2 atm) was performed during 30 min. Chemical poisoning with NaN_3_ was tested at a final concentration of 50 mM [8]. UV light irradiation was carried out during 3 h. Two microwave settings were tested to keep the samples below 70 °C: low power (LP; 160 W, 3 min) or medium power (MP; 500 W, 1 min) then cooled in dry ice (5 min). The heat-cold cycle was repeated 5 times. As the microwave technology can break down some plastic polymer, sterilization in PET bottles was not tested.

Effectiveness of sterilization procedure was monitored by optical microscopy using a Nikon® Eclipse E600 phase-contrast microscope. The strongest decrease in microbial cell counts was observed after heating (autoclave) or chemical poisoning (NaN_3_) for both methods in BG, FEP or PET bottles. Surprisingly, the lowest decrease in microbial cell counts was observed after the microwave treatments (LP and MP) in both BG and FEP bottles, while different studies reported the microwave efficiency to kill microorganisms [[9], [10], [11], [12]]. Depending on microwave treatment conditions, water molecules evaporate even faster when the volume of the sample is low. Our microwave settings were therefore adjusted to keep the samples (50 mL) below 70 °C in order to avoid evaporation. Unfortunately, these conditions were not sufficient to kill all the microorganisms. UV irradiation is a non-thermal alternative technology used for microorganisms inactivation by nucleic acids degradation [10,[13], [14], [15], [16]]. Cell damage may be temporary or permanent depending on the UV dose and the repair mechanisms developed by microorganisms. In this study, an efficient UV sterilization was observed in both BG and FEP bottles, contrary to the PET bottles, probably due to the UV filtering properties of the polymer which reduce the amount of UV and thus the DNA degradation.

**Fig. 3:**
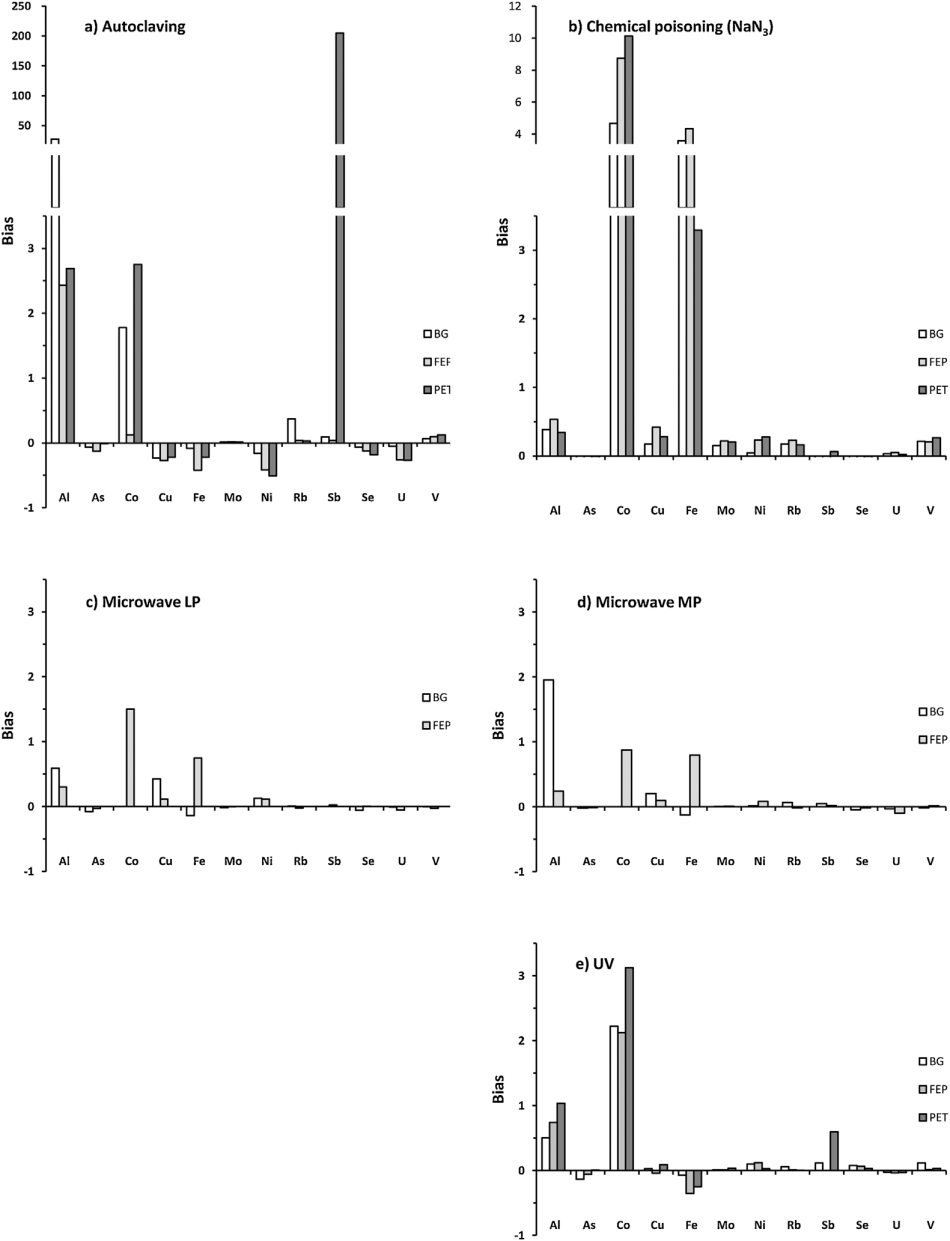
Impact of sterilization processes on chemical balances: (a) Autoclaving, (b) Chemical poisoning (NaN_3_), (c) Low Power (LP) microwaves, (d) Medium Power (MP) microwaves, (e) UV. Bias represents the normalized difference between trace metals concentrations after the sterilization protocol vs trace metals concentrations in the untreated sample (Control). Sterilization assays were performed in BG (white), FEP (light grey) and PET (dark grey) bottles. Trace metals with concentrations below the analytical limit of detection (Cd, Cr, Pb, Sc, Ti and Zn) are not presented.

Dissolved trace metals analyses were performed to study the impact of the sterilization protocols on chemical balances. After sterilization assays, samples were filtered using syringe filtration (filter porosity: 0.2 μm) in a cleanroom ISO 5. Trace metals analyses (Al, As, Cd, Co, Cr, Cu, Fe, Mo, Ni, Pb, Rb, Sb, Sc, Se, Ti, U, V, Zn) were performed by ICP-MS (Perkin Elmer®, Nexion 300X) in direct injection mode and details of analytical results are presented as additional information (Table S1). In order to compare the sterilization processes impact on chemical balances, results were normalized using the following equation:

$$Bias=\;\frac{{\left[M\right]}_{s}-{\left[M\right]}_{c}}{{\left[M\right]}_{c}}$$

where [M]_s_ and [M]_c_ represent the dissolved trace metals concentrations in the sterilized experimental and control bottles, respectively. A bias > 0 corresponds to a metallic contamination, whereas a bias < 0 represents metal scavenging due to complexation and/or precipitation processes. In this study, the strongest changes in dissolved trace metals concentrations were observed after heating (autoclaving, Fig. 3a) and chemical poisoning (NaN_3,_ Fig. 3b). This result is coherent with that of previous studies [3,[17], [18], [19]]. Although autoclaving is one of the most effective and cheapest sterilization methods used by microbiologists, this process modifies carbonates balances leading to the precipitation of metal hydroxides as well as modifications of the physicochemical characteristics of particulate matter. Indeed, the heat affects the behavior of organic matter that strongly increases the release of trace metals (e.g. Al, Co and Sb). Sodium azide (NaN_3_), an efficient respiratory inhibitor, is widely used in abiotic experiments but reduces nitrates-nitrites and changes pH leading to geochemical balances modifications [8,20,21]. Furthermore, the high concentrations of NaN_3_ (50 mM) required for sample poisoning can lead to significant contamination if the reagent is not trace metal free. In our tests, microwave treatments (LP and MP) had minor effects on geochemical balances (Fig. 3c and d), except for some elements with bias greater than 0.5 (e.g. Al, Co, Cr, Fe). However, the microwave settings were found insufficient to sterilize our samples. UV sterilization presented a similar geochemical pattern (bias lower than 0.5 except for Al, Co and Fe). Because UV irradiation was efficient for water samples sterilization in both BG or FEP bottles and presented minor changes in trace metal partitioning (Fig. 3e), this technique was further evaluated on several natural samples with or without sediments.

### Influence of UV irradiation on lacustrine samples

**Fig. 4:**
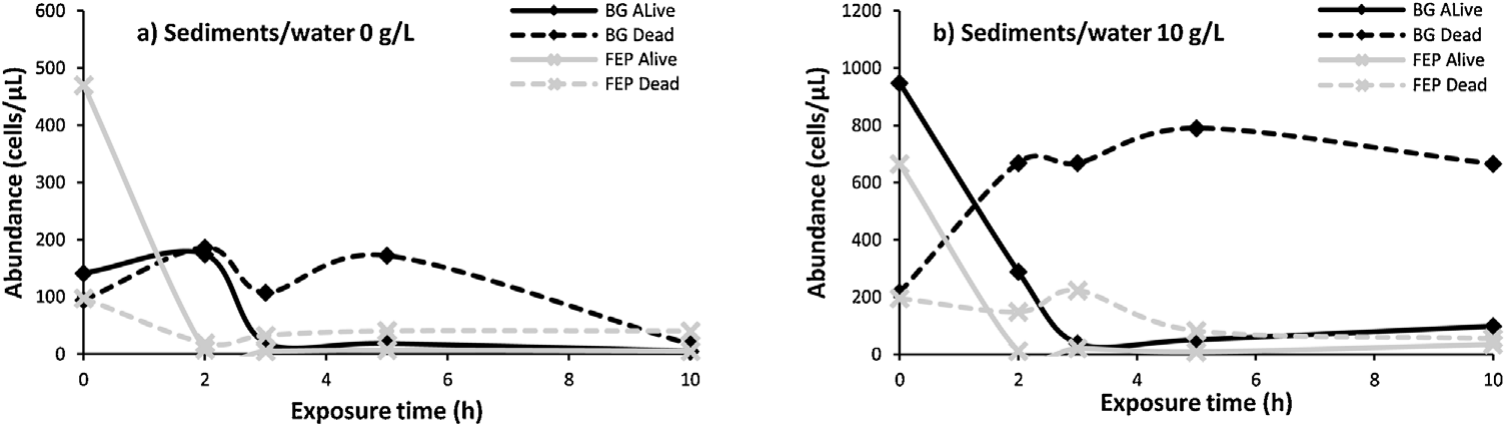
Variations of microorganisms activity (alive and dead cells) as a function of UV exposure time (0–10 h) in lacustrine samples (Peyrolles lake) in both sediments/water 0 g/L (a) and 10 g/L (b). Microcosms were performed using BG (black) and FEP (light grey) bottles.

Additional UV irradiation tests were carried out in lacustrine samples without or with sediments (0 or 10 g/L sediments/water conditions, respectively), in two types of bottles (BG or FEP). During 10 h-UV irradiation kinetic experiments, BG and FEP bottles were gently shaken manually every 30 min. The viable, damaged or compromised cells within free-bacterial community were quantified over time by epifluorescence microscopy with flow cytometry counting (BD Bioscience® FACSCalibur) according to the Nucleic Acid Double Staining (NADS) protocol [[22], [23], [24]]. Details of cells counting are presented as additional information (Table S2). For the 0 g/L sediments/water samples, a dramatic decrease in alive microbial cells was observed in both BG and FEP bottles after 10 h-UV irradiation tests (Fig. 4a). The abundance of alive cells decreased rapidly in the first 2 and 3 h (for FEP and BG bottles, respectively), then remained at a minimal level until the end of UV exposure. Dead cells abundance followed similar kinetic in FEP bottles, while they presented two relative maxima after 2 and 5 h in BG bottles before decreasing to the minimal level until 10 h of UV exposure. Similar results were obtained for the 10 g/L sediments/water samples (Fig. 4b). However, the alive cells abundance in BG appeared to increase slowly after 5 h of UV exposure, suggesting that a part of the cells population may have survived and grown in the sediments.

**Fig. 5:**
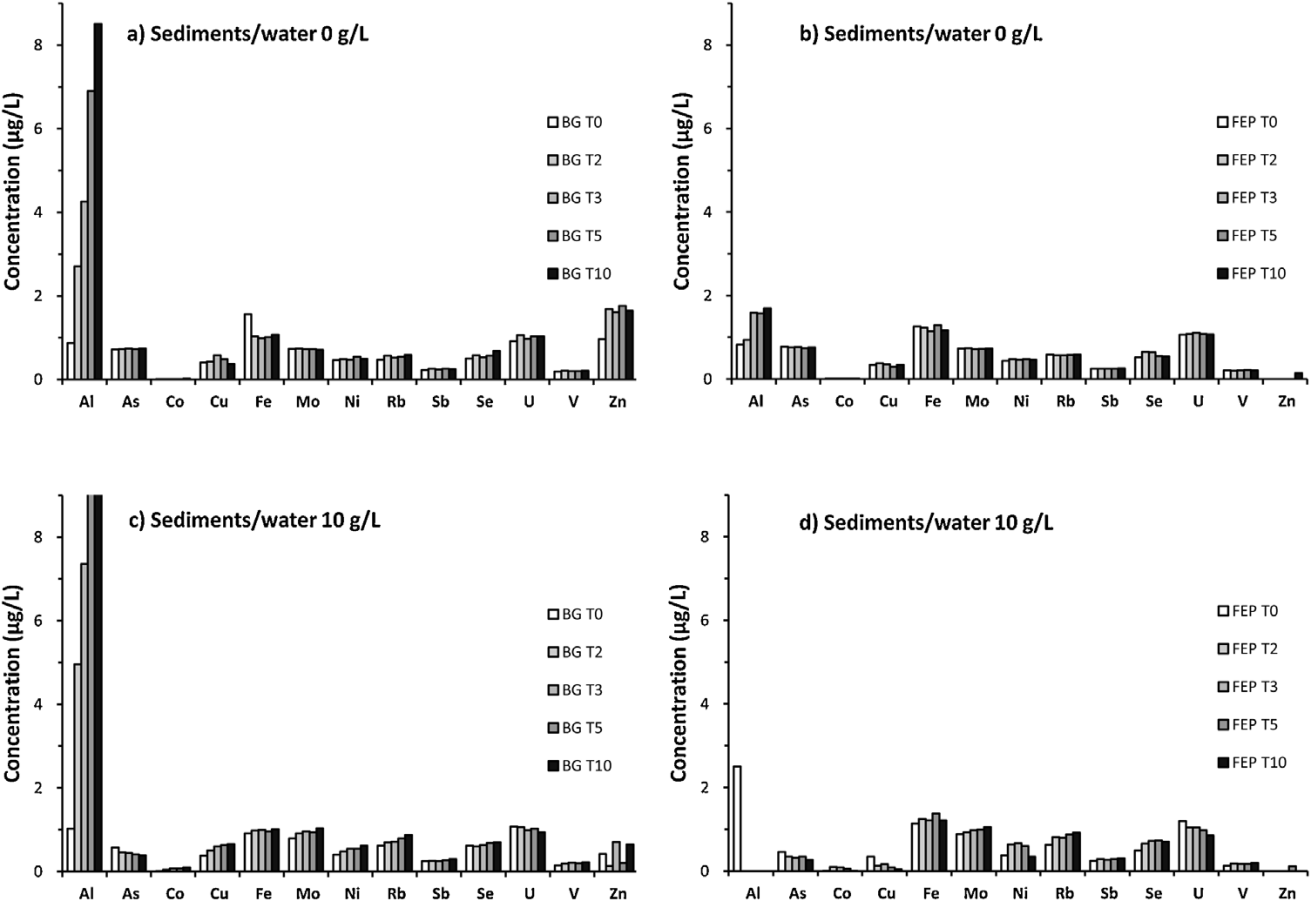
Variations of dissolved trace metals concentrations in lacustrine samples (Peyrolles lake) in both sediments/water 0 g/L (a and b) and 10 g/L (c and d). Samples were collected after five UV different exposure times: 0 h (T0), 2 h (T2), 3 h (T3), 5 h (T5), 10 h (T10). Microcosms were performed using BG (a and c) or FEP (b and d) bottles. Trace metals with concentrations below the analytical limit of detection (Cd, Cr, Pb, Sc and Ti) are not presented.

In order to assess the impact of UV exposure on geochemical balances, dissolved trace metals (Al, As, Cd, Co, Cr, Cu, Fe, Mo, Ni, Pb, Rb, Sb, Sc, Se, Ti, U, V, Zn) were also monitored over time (at 0, 2, 3, 5 and 10 h) for both 0 and 10 g/L sediments/water conditions, in BG and FEP bottles. Before ICP-MS (Perkin Elmer®, Nexion 300X) analyses, samples were filtered (0.2 μm) by syringe filtration in a cleanroom ISO 5. Details of analytical results are shown as additional information (Table S3). Some trace metals (Cd, Cr, Pb, Sc and Ti) with concentrations below the analytical limit of detection are not presented. In order to better assess the impact of UV irradiation on the concentration of each trace metal, the mean and dispersion of the analytical results were calculated:

$${\left[M\right]}_{m}=\frac{\sum_{i=T0}^{T10}{\left[M\right]}_{i}}{n}\pm \text{σ}$$

where [M]_m_ is the mean concentration, σ is the standard deviation and n the number of replicates. The dispersion indicators ([M]_m_ and σ) presented in Table 2 were calculated using analytical results of the kinetic of UV exposure (n = 5): 0 h (T0), 2 h (T2), 3 h (T3), 5 h (T5), 10 h (T10). For the 0 g/L sediments/water samples, both mean Al and Zn concentrations showed great discrepancies between the BG (4.65 and 1.53 μg/L, respectively) and FEP (1.32 and 0.14 μg/L, respectively) bottles, indicating that UV exposure and/or bottle material affect the distribution of these elements (Fig. 5a and b). Although the mean Fe concentrations were similar between the BG and FEP bottles (1.13 and 1.22 μg/L, respectively), the standard deviation for this element differs considerably (0.24 and 0.06 μg/L, respectively) which indicates a contamination during the BG T0 sample processing. By contrast, no difference between BG and FEP bottles was observed for the mean concentrations of the other trace metals (As, Co, Cu, Mo, Ni, Rb, Sb, Se, U, and V) with respect to UV exposure. However, standard deviations were consistently lower in FEP than BG samples, indicating that FEP bottles may prevent changes in trace metals partitioning. For complex matrices mixing water and sediments, the natural variations of the dissolved trace metals could overlap the variations related to the sterilization process, making the results interpretation ineffective. Indeed, for the 10 g/L sediments/water samples, the standard deviation (σ) of the trace metals mean concentrations were higher than for the 0 g/L samples, showing possible remobilization processes (Fig. 5c and d). No difference between BG and FEP bottles were observed for the mean concentrations of As, Co, Mo, Ni, Rb, Sb, Se, U, and V showing a conservative behavior with respect to UV exposure (Table 2). However, differences were observed in Cu (0.55 and 0.15 μg/L) and Fe (0.97 and 1.24 μg/L) concentrations in BG and FEP samples, respectively. Reduction of Cu^II^ to Cu^I^ and Fe^III^ to Fe^II^ in aquatic ecosystems occurs to a large extent via UV-induced photochemical reactions with organically chelated trace metals [25,26]. Then in the case of lacustrine ecosystems, the UV exposure of humic substances can impact the partitioning of Cu and Fe. Overall, the results obtained thanks to chemical and flow cytometry analyzes confirm that UV sterilization allows elimination of microorganisms respecting the distribution of trace metals (except Al, Cu, Fe and Zn) in lacustrine samples. The tests performed with FEP bottles allowed a faster sterilization and presented lower effects on trace metals concentrations than BG bottles.

**Table 2:** Dispersion indicators ([M]_m_ and σ; μg/L) of dissolved trace metals in the lacustrine samples calculated using analytical results of UV exposure kinetic (n = 5). Some dissolved trace metals (Cd, Cr, Pb, Sc and Ti) concentrations were below the analytical limit of detection.

	Sediment/water ratio 0 g/L				Sediment/water ratio 10 g/L			
	BG bottles		FEP bottles		BG bottles		FEP bottles	
	[M]_m_	± σ	[M]_m_	± σ	[M]_m_	± σ	[M]_m_	± σ
Al	4.65	± 3.09	1.32	± 0.41	8.49	± 6.69	2.50^a^	
As	0.73	± 0.01	0.76	± 0.01	0.45	± 0.07	0.34	± 0.10
Cd	<0.005		<0.005		<0.005		<0.005	
Co	0.009	± 0.004	0.005	± 0.003	0.056	± 0.035	0.049	± 0.043
Cr	<0.05		<0.05		<0.05		<0.05	
Cu	0.45	± 0.08	0.34	± 0.03	0.55	± 0.11	0.15	± 0.12
Fe	1.13	± 0.24	1.22	± 0.06	0.97	± 0.04	1.24	± 0.09
Mo	0.72	± 0.01	0.73	± 0.01	0.92	± 0.09	0.96	± 0.06
Ni	0.49	± 0.03	0.46	± 0.02	0.51	± 0.08	0.52	± 0.15
Pb	<0.005		<0.005		<0.005		<0.005	
Rb	0.54	± 0.04	0.58	± 0.01	0.74	± 0.09	0.80	± 0.11
Sb	0.24	± 0.02	0.25	± 0.01	0.26	± 0.02	0.27	± 0.02
Sc	<0.05		<0.05		<0.05		<0.05	
Se	0.57	± 0.07	0.58	± 0.06	0.64	± 0.04	0.66	± 0.10
Ti	<0.5		<0.5		<0.5		<0.5	
U	0.10	± 0.06	1.08	± 0.02	1.01	± 0.06	1.02	± 0.13
V	0.20	± 0.01	0.21	± 0.01	0.19	± 0.03	0.17	± 0.02
Zn	1.53	± 0.32	0.14^a^		0.42	± 0.26	0.11^a^	

a n = 1.

### Validation of the UV sterilization protocol with coastal marine samples

The influence of UV irradiation was also considered in batch experiments conducted in triplicate (n = 3) on coastal marine samples without or with sediments (sediments/water 0 or 10 g/L, respectively), in two types of bottles (BG or FEP). According to the results obtained on lacustrine samples, a 4 h-UV irradiation was chosen to achieve a complete sterilization and compared to a biotic control (UV exposure: 0 h). During tests, BG and FEP bottles were shaken manually every 30 min. The samples stability was also monitored after 4 cycles of UV exposure (4 h UV/24 h).

**Fig. 6:**
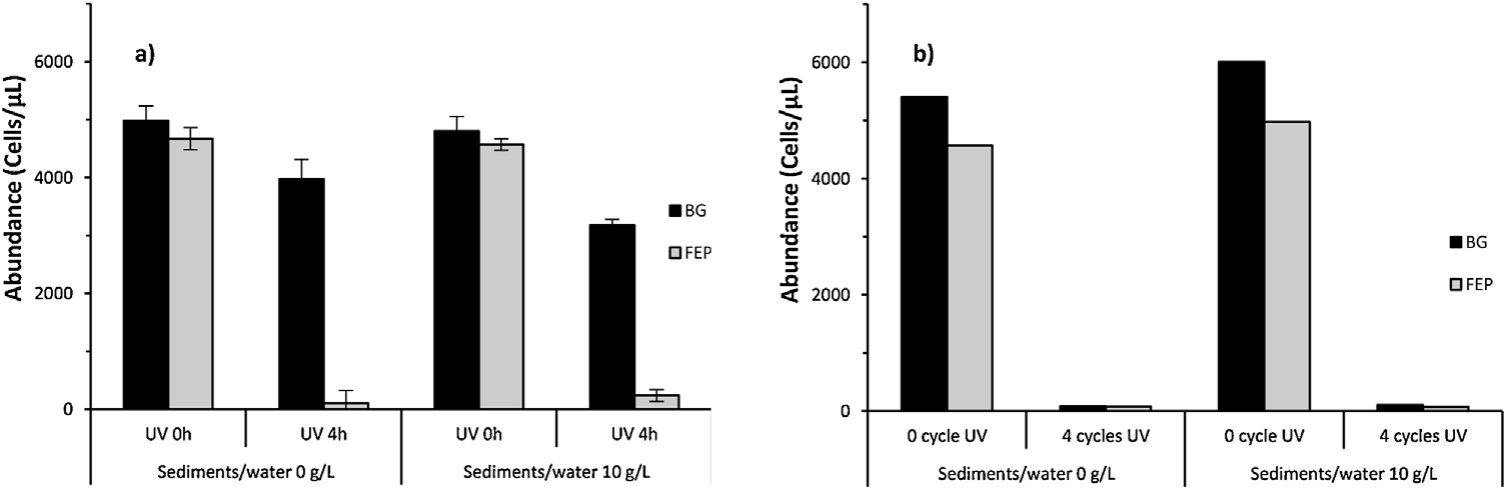
Variations of microorganisms viability (alive cells) in coastal marine samples (Bay of Marseille), in both sediments/water (0 and 10 g/L) and exposed to two UV irradiation time: 0 or 4 h (a), and 0 or after 4 cycles repeating 4 h at interval of 24 h. Microcosms were performed using BG (black) or FEP (light grey) bottles. For 0 and 4 h-UV irradiation time (a), values are means (n = 3): cells counting ± standard deviation (error bars).

The quantification of viable, damaged or compromised cells within the free-bacterial community was also assessed by epifluorescence microscopy with flow cytometry counting (BD Bioscience® FACSCalibur) using the NADS protocol [[22], [23], [24]]. The details of cells counting are presented as additional information (Table S4). Very reproducible results were obtained whatever the bottle type and sediments/water condition used (Fig. 6a). However, concerning the effectiveness of UV sterilization, differences were observed between BG and FEP bottles. Indeed, a 4-hours UV exposure was efficient to eliminate the most of the microorganisms in FEP bottles (98% and 95% of cells mortality for 0 and 10 g/L sediments/water conditions, respectively), but was not effective in BG bottles (20% and 34% of cells mortality for 0 and 10 g/L sediments/water conditions, respectively). However, the large majority of live cells was eliminated (>98% loss) in both BG and FEP bottles from both 0 and 10 g/L sediments/water conditions by repeating this batch experiments with 4 cycles of UV exposure (4 h UV/24 h) (Fig. 6b).

**Fig. 7:**
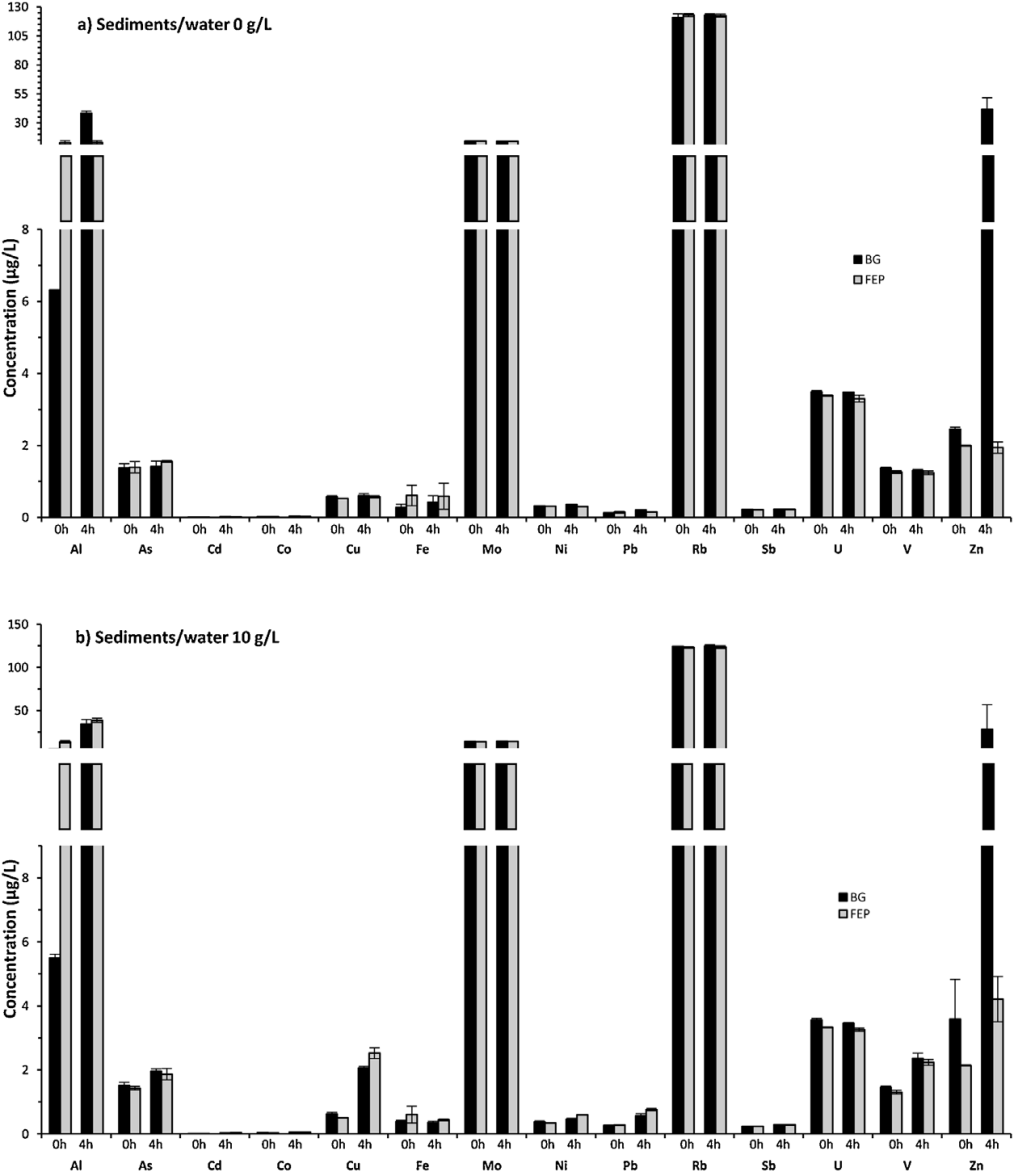
Variations of dissolved trace metals concentrations in coastal marine samples (Bay of Marseille), in both sediments/water 0 g/L (a) and 10 g/L (b) and exposed to two UV irradiation time: 0 h or 4 h. Microcosms were performed using BG (dark grey) or FEP (light grey) bottles. Values are means (n = 3): trace metal concentration ± standard deviation (error bars).

**Fig. 8:**
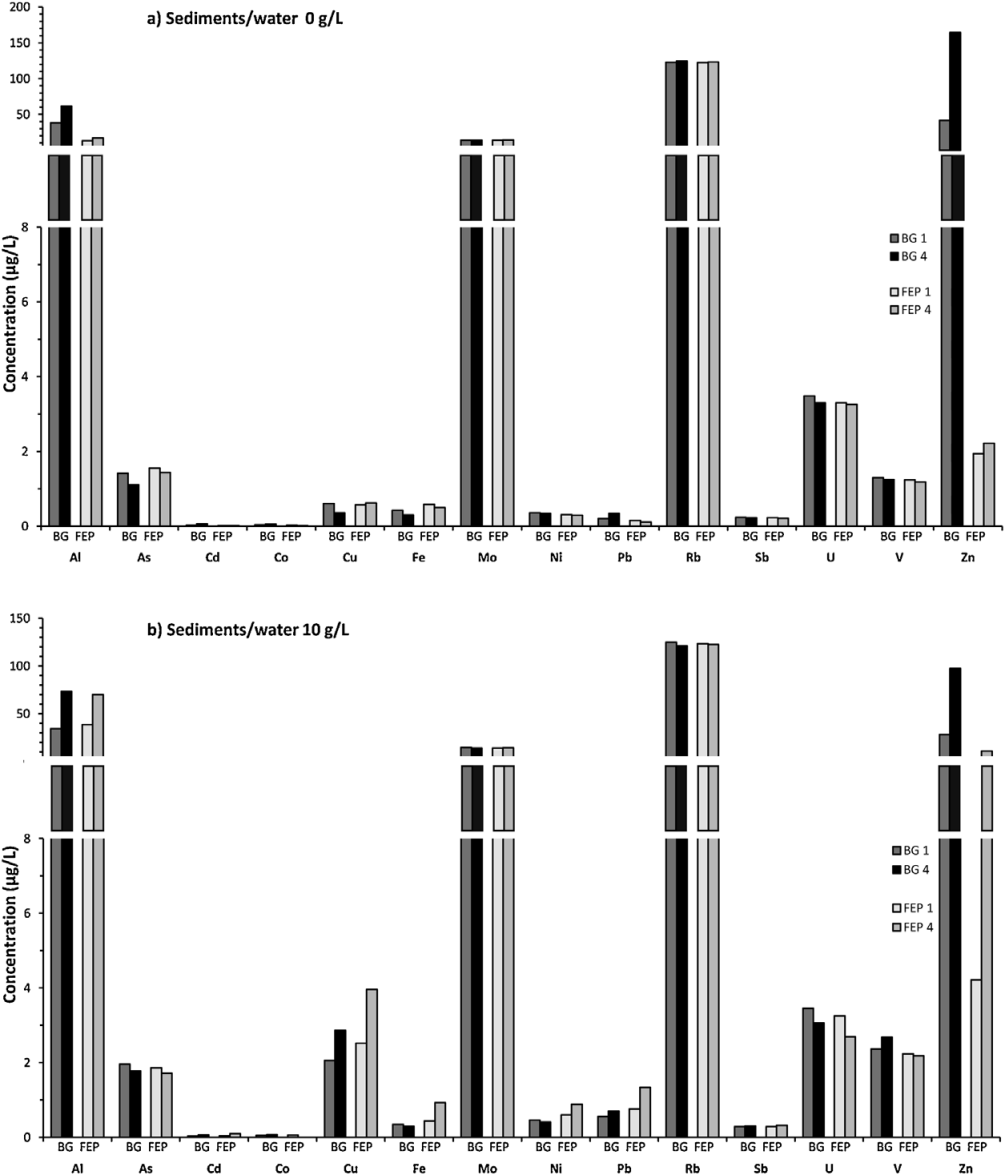
Variations of dissolved trace metals concentrations in coastal marine samples (Bay of Marseille), in both sediments/water 0 g/L (a) and 10 g/L (b) and exposed to two UV irradiation sequences: 1 or 4 cycles repeating 4 h at interval of 24 h. Microcosms were performed using BG (dark grey) or FEP (light grey) bottles.

Analyzes of dissolved trace metals (Al, As, Cd, Co, Cr, Cu, Fe, Mo, Ni, Pb, Rb, Sb, Se, U, V, Zn) were also performed on these tests to assess the impact of UV exposure on geochemical balances. After UV irradiation, samples were filtered (0.2 μm pore size) by syringe filtration in a cleanroom ISO 5. Trace metals analyzes were carried out by Argon Gas Dilution Inductively Coupled Plasma Mass Spectrometry (AGD-ICP-MS; Thermo Scientific®, iCAP-Q) in direct injection mode. AGD is a useful automatic technique to reduce matrix loading on cones and nebulizer. Details of analytical results are shown as additional information (Table S5). Trace metals with concentrations below the analytical limit of detection (Cr and Se) are not presented here. The repeatability of sterilization process was evaluated with triplicates (n = 3) and differences in trace metals concentration between BG and FEP bottles were compared after 0 and 4 h-UV irradiation, in both 0 and 10 g/L sediments/water conditions (Fig. 7a and b, respectively). Except for Al, Fe and Zn, concentrations of dissolved trace metals were similar between BG and FEP bottles (variability < 10%). Differences in Al, Fe and Zn concentrations between BG and FEP bottles indicate a possible impact of UV exposure and/or bottle material on geochemical properties of these elements. Other elements (As, Cd, Co, Cr, Cu, Mo, Ni, Pb, Rb, Sb, Se, U, V) appeared little or not affected by UV irradiation in these conditions. However, it is difficult to estimate the impact of UV exposure on geochemical balance of particle-rich samples, as natural processes can also induce significant variations in the distribution of dissolved trace metals. Similar dissolved trace metals concentrations were observed in coastal marine samples (sediments/water 0 g/L) after 1 cycle (4 h) and 4 cycles (4 h UV/24 h) showing that repeated irradiation does not alter the water samples (Fig. 8a). Regarding the complex 10 g/L sediments/water samples, the variability of dissolved trace metals increased after 4 cycles of 4 h-UV exposure (sediments/water 10 g/L) (Fig. 8b). Thus, further investigation should be conducted to assess the impact of repeated UV exposure on the distribution of trace metals between the solid and liquid phases.

## Conclusion

Unlike other methods, UV sterilization presented minor effects on the distribution of dissolved trace metals. UV irradiation on lacustrine samples showed faster sterilization and lower variations of dissolved trace metals concentrations (except for Al, Cu, Fe and Zn) using FEP bottles than using BG bottles. We recommend 3 h of UV irradiation for maximum efficiency. For coastal marine samples, UV sterilization were obtained on both 0 and 10 g/L sediments/water, in BG and FEP bottles. UV exposure of 4 h was efficient to eliminate the majority of microorganisms in FEP bottles, while this exposure time was not effective to sterilize in BG bottles. We recommend a repeated 4 cycles of UV irradiation (4 h UV/24 h) for maximum efficiency. However, it is difficult to estimate the impact of repeated UV exposures on the geochemical balance of marine particle-rich samples.

## Additional background

Sterilization techniques can be classified into four categories: heat, filtration, chemical and electromagnetic wave. Each technique has its benefits and limitations, and no detail focusing on the trace metal balances have been reported until now. Heat sterilization (e.g. autoclaving, pasteurization, tyndallization) is a widespread technique used to kill microorganisms. Autoclaving generates high pressure and temperature conditions (2 atm, 121 °C) preventing liquid evaporation. However, these drastic conditions induce an increase of pH and a loss of CO_2_ leading to chemical balances modifications [27]. Pasteurization and tyndallization are two other heat methods used to avoid autoclaving artefacts but are less efficient to kill the microorganisms [28]. Filtration is also an alternative sterilization technique used for liquids with heat-labile components, but is not appropriate for complex samples (matrices mixing water and sediments). Many chemicals reagents (e.g. HgCl_2_, NaN_3_) can be used for sterilization; however, these reagents alter physicochemical characteristics and may introduce contaminants in the sample [3,20]. The electromagnetic waves (e.g. microwave, UV irradiation) are used as alternative sterilization techniques for materials (sample, recipient) sensitive to thermal effects. Microwave does not alter pH and avoid the inorganic compounds precipitation but can damage plastics recipients and induce heat effects [9,29].

UV radiations are spread on a wide electromagnetic spectrum subdivided into four classes: i) UV-A from 400 to 315 nm, ii) UV-B from 315 to 280 nm, iii) UV-C from 280 to 200 nm and, iv) vacuum UV from 200 to 100 nm. The best sterilization with UV is generally obtained using UV-B and UV-C, with a maximal effect at 254 nm [15]. UV-C consist in very high energy wavelengths but as such do not penetrate very deeply into the sample as they interact quickly with the matter, limiting sterilization to surfaces or transparent materials [30]. The bactericidal effect of UV-A is low compared to UV-B and UV-C, but a longer exposure time may have a sterilizing action [31]. Finally, vacuum UV also have a sterilization action but remain unsuitable because these very short wavelength dissipate too rapidly in water, over very short distances [32].

Mercury lamps are commonly used light sources for samples sterilization due to its UV emissions in the bactericidal wavelength range. Here, we used a medium-pressure polychromatic mercury lamp (Philips®, HPL-N 125W) whose light bulb was carefully removed in order to prevent any absorption of UV wavelengths across the glass wall. Furthermore, our homemade UV irradiation chamber was lined inside of aluminum foil used to reflect and thus concentrate UV light and increase its intensity (Fig. S1 as Supporting information). Finally, a ballast (ELT®, VMI 12/23-3) was used to regulate the incoming power supply at the level needed to energize and operate the UV lamp. The UV chamber designed in this study can be easily used to sterillize several complex samples at the same time, without disturbing their chemical composition.
