# Supplementary material for: Impact of sterilization methods on dissolved trace metals concentrations in complex natural samples: Optimization of UV irradiation
Source: MethodsX. 2019 Apr 22;6:1133–46. doi: 10.1016/j.mex.2019.04.020 (PMC6529719; doi:10.1016/j.mex.2019.04.020)
Supplement: Supplementary file 1 [file mmc1.pdf]

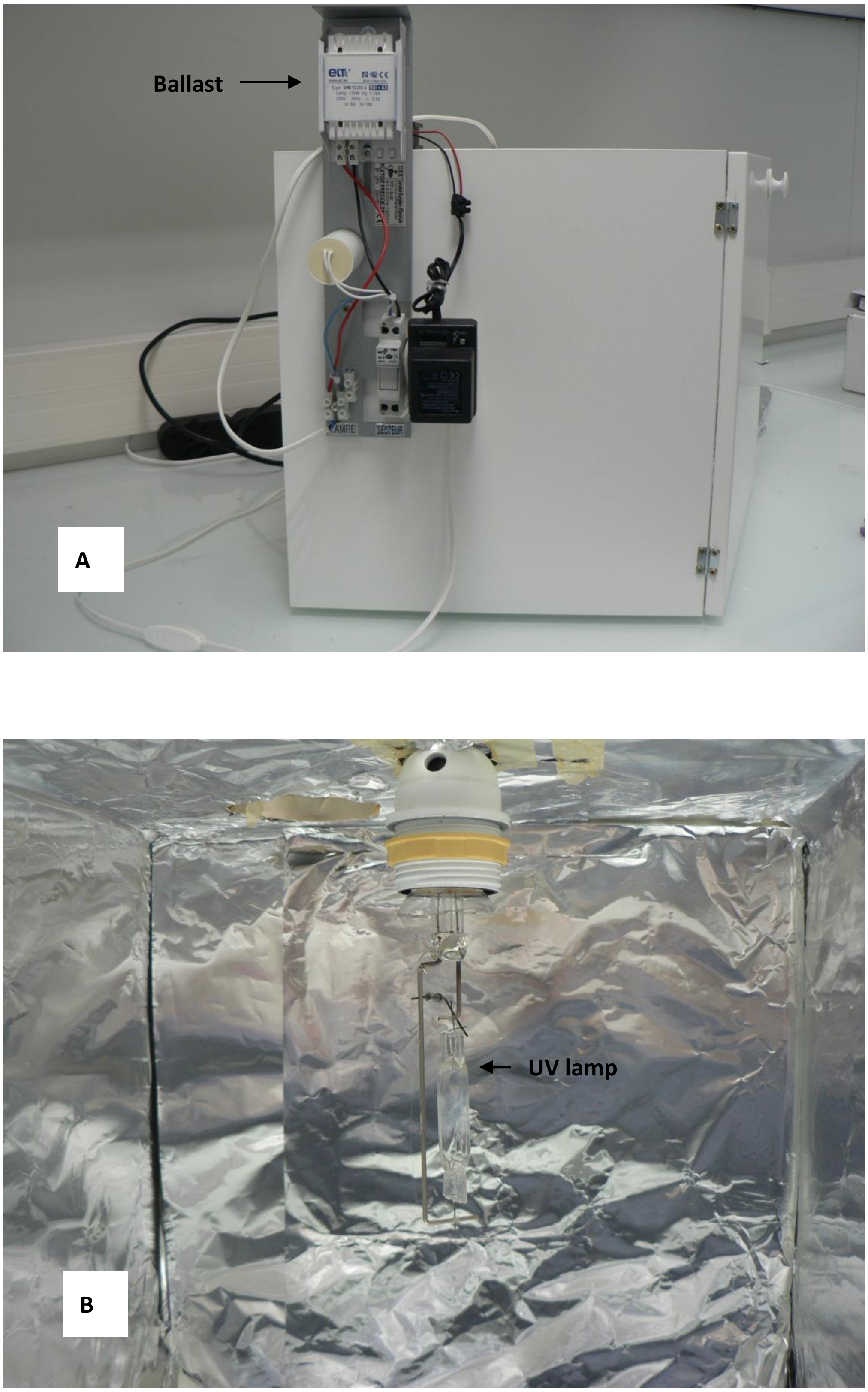

Fig S1: Home-made UV irradiation chamber. A) Outside view showing ballast connexion, B) inside view showing UV lamp
